# Ileostomy Adenocarcinoma After Recurrent Rectal and Colon Cancer: A Case Report and Literature Review

**DOI:** 10.7759/cureus.96284

**Published:** 2025-11-07

**Authors:** Joseph Chi Lok Lo

**Affiliations:** 1 Surgery, Caritas Medical Centre, Hong Kong, HKG

**Keywords:** ileostomy carcinoma, ileostomy complications, stoma malignancy, stoma recurrence, surgical case reports

## Abstract

Numerous complications of stoma have been documented in the literature, yet only a few involve malignancy. However, missing or delayed diagnosis can lead to disease progression, metastasis, and death. We present a case of a 71-year-old patient who had a history of recurrent colorectal cancer with multiple operations, including an end ileostomy, who suffered from an ileostomy adenocarcinoma, presenting with abnormal growth on the mucocutaneous junction of the stoma, in the absence of high-risk features, including familial adenomatous polyposis or inflammatory bowel disease. Diagnosis was confirmed with biopsy, and the patient underwent surgical excision with a satisfactory outcome.

There are previous case reports and studies on ileostomy carcinoma, which suggest the incidence, typical presentation, risk factors, pathophysiology, and treatment methods. This case illustrated the possibility of rare stoma malignancy in a patient with a relatively short stoma age and absence of high-risk predisposing conditions. Ileostomy carcinoma is a rare complication, but clinicians should be highly alert to new symptoms of the stoma and take biopsies of any suspicious lesions, as early detection and surgical excision can lead to a favorable prognosis.

## Introduction

Ileostomy is a common procedure performed in general surgery, followed by bowel resection. Complications related to stomas are common, with most being benign in nature, including prolapse, retraction, stenosis, parastomal hernia, skin irritation, and infection. However, from time to time, there are reports of malignancy occurring in stoma sites, but no standard guidelines are available due to their low incidence. The following illustrates another case related to this sinister pathology, but with a significant difference regarding the incidence itself and the patient’s characteristics.

## Case presentation

A 71-year-old lady, who was a chronic smoker, was first diagnosed with Duke’s C adenocarcinoma of the rectum in 1992, with a low anterior resection done. The margin was clear, and she underwent adjuvant chemotherapy. Five years later, she was found to have a recurrence of rectal adenocarcinoma alongside a synchronous tumor at the cecum. She subsequently received abdominal perineal resection and right hemicolectomy in the same session, with an ileocolic anastomosis and an end colostomy. The margin was clear, and adjuvant chemoradiation therapy was administered. Surveillance colonoscopy in 2002 confirmed no recurrence.

**Figure 1:**
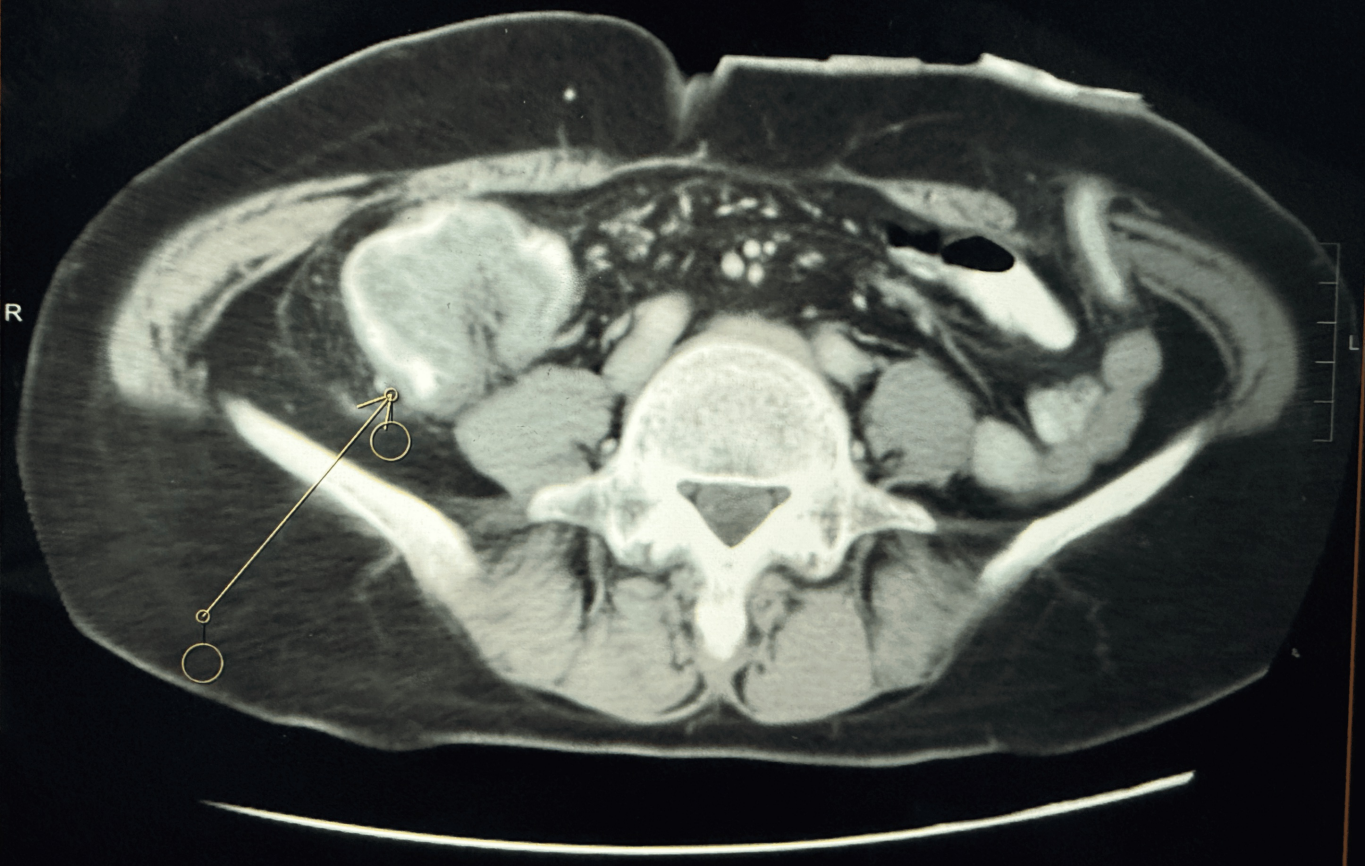
Computed tomography showing 4.5 cm right side CA colon. The arrow points to the recurrent mass. CA: adenocarcinoma

Afterwards, the patient had half yearly to annual follow-up for her condition. In 2006, she complained of lower abdominal pain with some weight loss. A colonoscopy was done, showing a large fungating mass at 50 cm, with biopsy confirmed adenocarcinoma. Computed tomography revealed a 4.5 cm mass on the right side of the residual transverse colon with no distant metastasis (Figure 1). A completion total colectomy followed by end ileostomy was performed, with disease staging to be Dukes B, pT3N0, margin clear. No adjuvant therapy was given.

**Figure 2:**
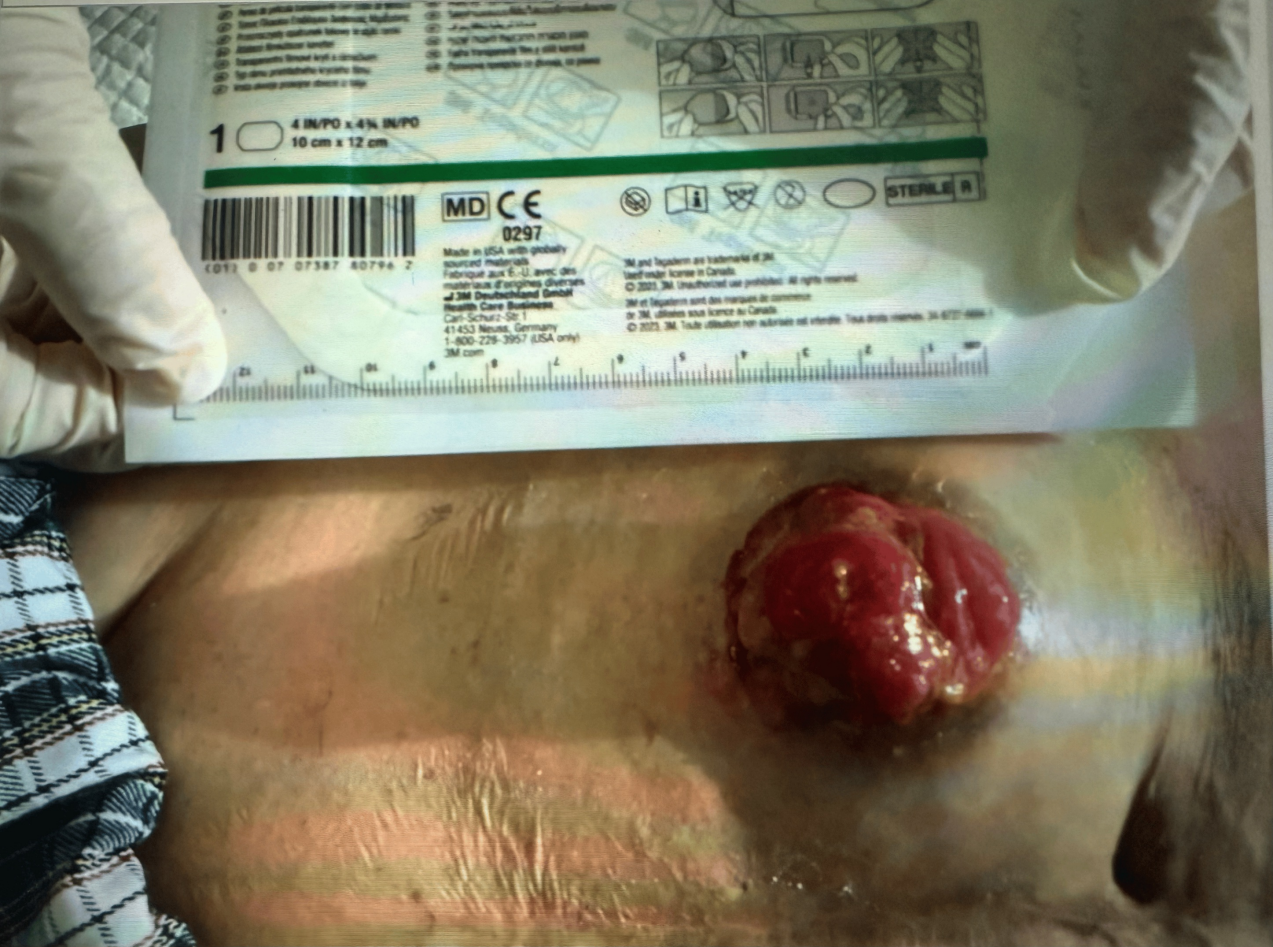
Irregular growth on ileostomy with biopsy-confirmed adenocarcinoma.

The patient was initially advised to undergo half-yearly follow-up for the next five years, then transitioned to regular yearly or alternate-year follow-up, during which the stoma condition was assessed as unremarkable. The latest follow-up session took place in June 2024. In March 2025, she was admitted to the hospital for abdominal pain and blood-stained discharge from the distended stoma. Physical examination found irregular growth over lateral side of stoma at the mucocutaneous junction (Figure 2). A biopsy done at the bedside confirmed adenocarcinoma. Carcinoembryonic antigen (CEA) level was normal upon diagnosis (reference range <3 for nonsmokers, <5 for smokers). Computed tomography showed a 2.6 cm outpouching at the ileostomy with adjacent skin thickening; otherwise, there was no distant metastasis. Surgical management consisted of wide local excision of the stoma with small bowel resection of 5 cm from the tumor, as well as excision of a 2 mm polyp 1 cm from the proximal margin. Skin margin of 5 mm was excised, and a new end ileostomy was then created.

**Figure 3:**
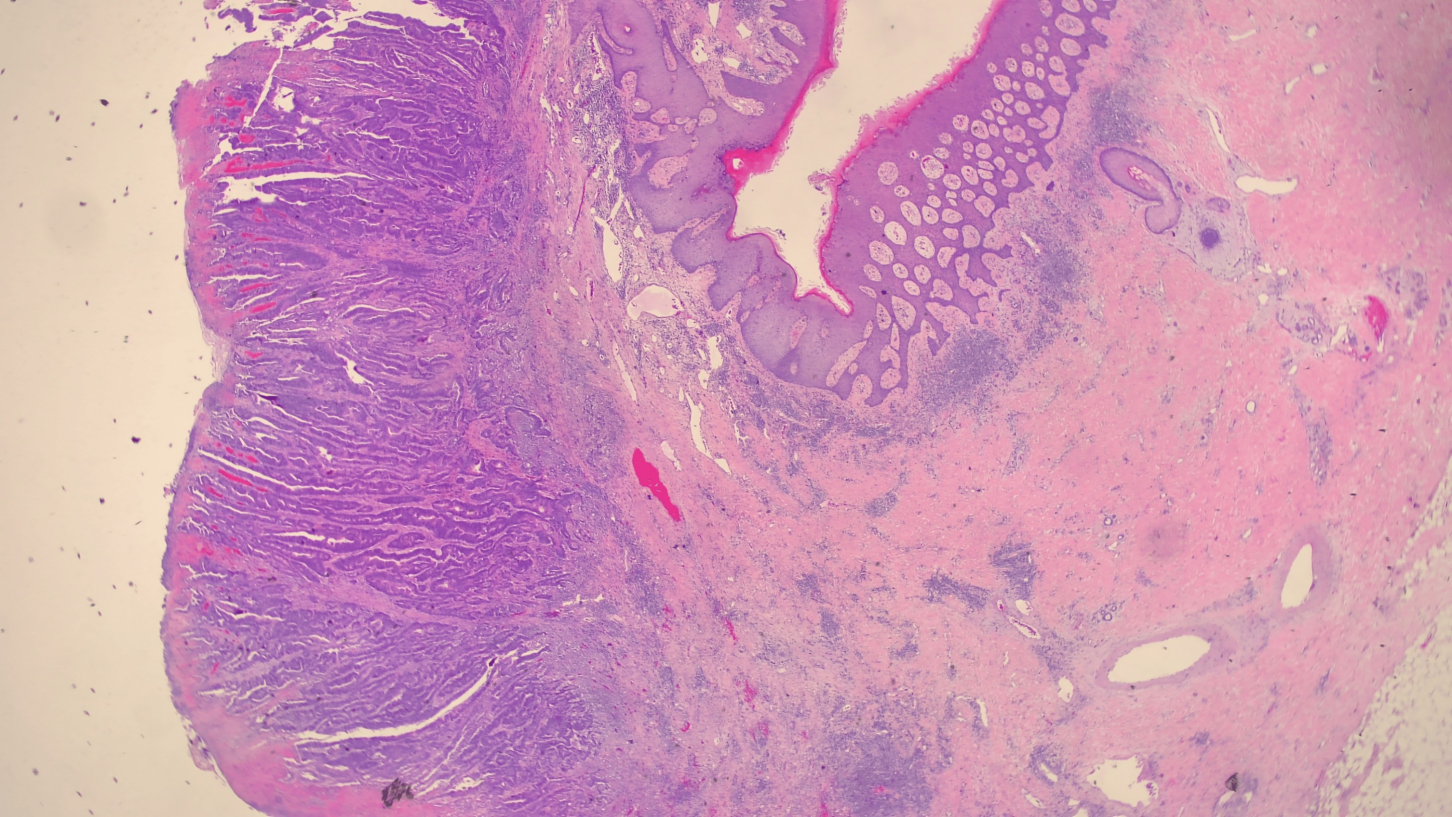
Ileostomy carcinoma, low power.

**Figure 4:**
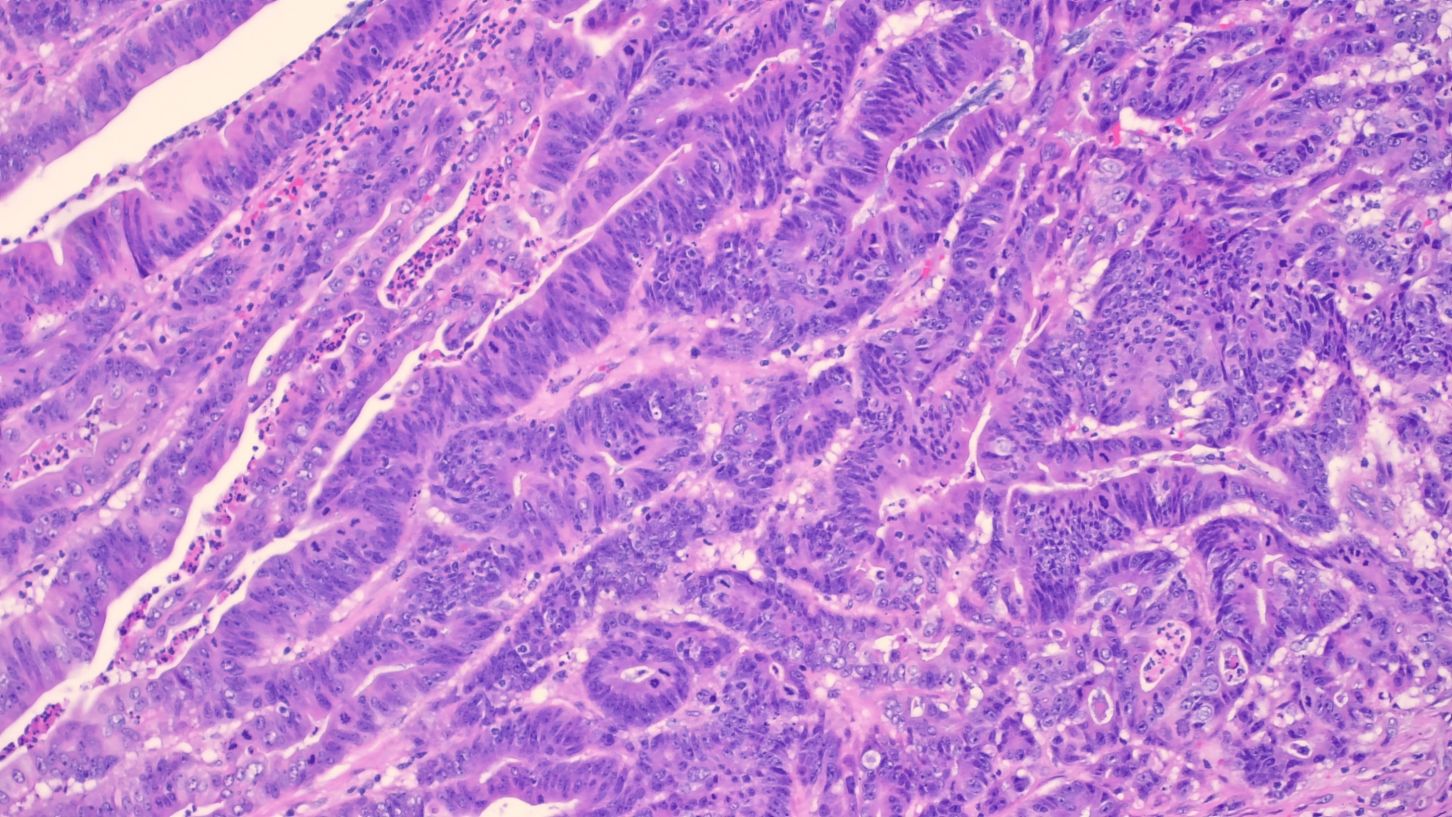
Ileostomy carcinoma, high power.

In the early postoperative phase, the patient suffered from high output from the stoma and was treated with loperamide and intravenous fluid for hydration. She was also given stomahesive paste to minimize skin irritation, and eventually discharged on postoperative day 10 after removal of stitches. There were otherwise no major complications. The pathology of the specimen revealed moderately differentiated adenocarcinoma with clear margins and no nodal metastasis, as well as polypoid mucosa in the resected polyp (Figures 3, 4). She was advised to undergo a three-month follow-up and surveillance contrast-enhanced computed tomography a year later.

## Discussion

This case described the occurrence of ileostomy cancer in a patient with no known genetic condition predisposing to bowel malignancy at a relatively short stoma age. Ileostomy carcinoma is a rare but potentially life-threatening condition associated with the creation of an ileostomy. There have been 91 cases reported as of 2020, with indications of ileostomy creation mainly for inflammatory bowel disease and familial adenomatous polyposis [1]. Adenocarcinoma (76.9%), followed by squamous cell carcinoma (11%), were the most common ileostomy site cancers.

The etiology of ileostomy adenocarcinoma is unclear, with various hypotheses. The most popular one suggested that chronic irritation, both physically from trauma and chemically from adhesives used in conjunction with stoma appliances, instigates epithelial oncogenesis, giving rise to metaplasia, dysplasia, and malignancy [2]. Moreover, the presence of bacterial flora in patients with long-standing ileostomies resembling in bacterial type and concentration that of the normal colon rather than that of the normal ileum, as well as the finding of colonic type of mucin in the metaplastic mucosa of ileostomies, adds support to the theory of metaplasia in ileal mucosa [2,3]. Familial adenomatous polyposis (FAP) is a major risk factor for ileostomy carcinoma. Patients with ileostomy after proctocolectomy are prone to developing ileal adenomas, which can evolve into carcinoma [4]. Inflammatory bowel disease (IBD) is also another risk factor. On average, malignancies developed 29.4 years after ileostomy creation. It was also shown that FAP patients developed ileostomy site malignancies at a younger age and had a shorter stomal duration [1].

Patients usually present with bleeding, difficulty in fitting a stoma appliance, obstruction, and an incidental finding of a polypoid mass or ulcer. A biopsy should be performed on a suspicious lesion in the ileostomy to confirm the diagnosis and to exclude other differentials, including ileitis or backwash ileitis at the stoma, Crohn’s, pseudoepitheliomatous hyperplasia, polyps, granulation tissue, or pyoderma gangrenosum, which are more common conditions found in the ileostomy. A biopsy should be taken, especially for newly developed lesions. Ileoscopy can serve as an adjunct for mucosal inspection [5].

An en bloc wide local excision of the ileostomy, including the adjacent anterior abdominal wall, with or without transposition of the stoma to a new site, has been shown to provide the best prognosis for an adenocarcinoma arising from an ileostomy, with an 85% survival rate [6,7]. Adjuvant therapy may have a benefit if there is lymph node involvement.

## Conclusions

Ileostomy carcinoma is a rare condition but clinicians should be highly alert to new symptoms on stoma and take biopsies for any suspicious lesions, as overall prognosis is good with surgical excision. Patients should receive regular surveillance of stoma condition for early diagnosis.
